# The Old and the New in Dentistry

**Published:** 1900-05-15

**Authors:** C. R. Rowley


### THE OLD AND THE NEW IN DENTISTRY.

BY C. R. ROWLEY, D.D.S.

Read before the Southwestern Michigan Dental Society, April 10, 1900.

*Mr. President, Ladies and Gentlemen:*—It would be presumptuous on my part, to even attempt to speak of any thing New in the practice of dentistry, to a body of men constituting the membership of this society. As I take it, you are as well, if not better acquainted with the modern appliances and methods of operating as I am. And equally presumptuous to speak of the Old. But as the welfare of a dental society depends somewhat largely upon the number of papers presented and not necessarily on their quality, I ventue this contribution, and having the upbuilding, and perpetuity of this particular organization at heart, I am moved to present you a paper—though it may count in quantity only—and to obey the commands of your Executive Committee, in its request to present a paper at this meeting.

In speaking of the Old we wish to turn back the pages of history no farther than exists in the memory of our oldest practictioners, neither will we speak disparagingly nor critically of the operations, or methods of practice adopted by and used by our fathers. The trend of this paper will be more to enter a comparison, and thereby see how far we have drifted.

The young and progressive dentist of to-day looks back historically two or three decades, and concludes, the dental profession has emerged from the slums or dark ages; whereas, in fact, there are only words of commendation to be said or written of the Old in Dentistry. We have the defenders of past methods, who point with pride to the soft gold fillings made without the aid of a rubber dam, clamp or mallet. So soft a pin could be driven from periphery to base, and yet the filling remaining serviceable many times longer than the five years now alloted to the average gold filling.

They tell of the carefull cavity preparation by hand, without display of machinery, to frighten the timid, or excessive destruction of enamel walls by rapidly revolving bur. They tell us of tin so compatible to tooth structure and conducive to oral hygiene—a theory admitted by the New in Dentistry to-day.

In prosthetic destistry, they guide us to a laboratory where the beautiful continuous gum, gold and silver plates are made. Where the various parts of cleft palate apparatus are made and put together before going to the operating room for adjustment. Where the delicate bands and bars, used in orthodontia are made with precision and skill equally great as any attempted by the modern dentist. With these facts before us, who can truthfully say that dentistry was groping in the dark, or walking in the bypaths of scientific investigation, forty or fifty years ago. Who can gainsay the fact, that dentistry of Old required equally as great a degree of proficiency, as it does this tenth day of April, 1900. In studying the men in practice, forty and fifty years ago, we will see that their skill and proficiency enabled them to charge prices commensurate with services rendered, giving them a good balance on the right side of the ledger at the close of every year. Without dental colleges, excepting two, possibly three; without dental faculties, examining boards, or state laws. The profession of dentistry forty-five years ago, and even beyond that period, stood in her own class; seeking no recognition from any other learned body, and yet demanding and receiving respect and attention from all skilled artisans. Only men of unquestioned ability sought admission to the profession, and these were compelled to serve a long apprenticeship before mastering the technicalities they encountered. In this we have a glimpse of dentistry when the advent of rubber for base plates came as a pestilence to sicken, and a cloud to darken, and yet as a great blessing in disguise. With the coming of rubber, thousands rushed in where but few had dared to enter. So great was the influx, and so easy the acquirement of the method, that state laws became a necessity to protect the people from injury from practitioners professing to be dentists, after serving a thirty day apprenticeship. The law demanded a higher standard, hence the wonderful development of dental colleges, until, to day, we have one for about every state in the Union. And, just here let us ask, and briefly answer: What are some of the things the dental colleges have done for the men and women practicing dentistry?

First, they have given us a degree, and title, and we are known as Doctors of Dental Surgery; a distinction enjoyed by nine-tenths of the dentists now in practice, and to be eventually by all, as the future will not recognize any one without a title. They have made dentistry a specialty in practice of medicine, by giving students the same course of studies, with a few exceptions, exacted by the medical schools. They have, to the man or woman seeking admission to dentistry, given *all* possible for them to attain. They have to a large degree, impressed the public by their strength and dignity, that a newly made graduate is better qualified to care for their teeth than the man of experience. They have retained and enlarged upon all that was good in the past, and are ever seeking for something better in the future. They are saying to the people, there will soon be a passing away of state laws, as the graduate of their temples of learning will carry with them a passport in their diploma, to the entire satisfaction and confidence of the people. And now, with your indulgence, I would like to speak of the Old in Dentistry as compared with the New.

So far as I am able to judge, anatomy is taught the same to day as it was fifty years ago. The muscles, bones, nerves and teeth are the same. Pronunciation has changed some, which I will mention later. In anatomy, the freshman of the past, down to the present time, soon memorized *Lavator Labii Superioris, Alæque Nasi Muscle*, and verbally hurled it in the face of his friends that he might display his great learning in anatomy.

The eye, or stomach teeth, now known and taught as the cuspids were in the Old known by their Latin names, *cuspidati*. Dentin was always pronounced denteen. Interdental space was as the approximal or inbetween. The words gingival, mesial and distal are not found in our old text books. Instead, however, a sufficency of angles are mentioned. We read of the anterior or posterior bucco-approximal, and the anterior or posterior palato angles. These terms are not used by the New in Dentistry to-day.

Arsenic was used for destroying pulps fifty years ago, the time allowed for action was six and seven hours; whereas now we believe it safe to allow as many days. The actual cautery was quite generally used, driving a heated wire as far into the nerve canal as possible. We also find mention of the immediate removal of the pulp and nerve with the broach, without any previous preparation or treatment, and all the writers tell us, with little if any pain.

The rubber dam did not come into general use until about the year 1873. The dental engine arrived at about the same period. The automatic mallet was in use a number of years prior to the dam or engine. Harris says, in his Principles and Practice of Dental Surgery, sixth edition, "That just in proportion as the dentinal structure of the teeth is hard or soft, their shape perfect or imperfect, their arrangement regular or irregular, is the liability to caries diminished or increased." This theory has been proven by the New in Dentistry to be incorrect. Ten pages of the same book are devoted to filling the teeth and securing room for the proper insertions of fillings, as a prevention of decay. Decay of the teeth or caries was supposed to originate by some corrosive substance getting under the enamel, how it got there is not mentioned—working into the dentin until the tooth was destroyed. The New in Dentistry believe in the germ theory. The bachteria building for their habitat a plaque shaped house, upon a part of the tooth, generally away from the encroachments of brush or other interference, where at leisure by secreting a destructive substance which attacks readily the enamel and more readily the dentin. This theory would have been objectionable to the Old in Dentistry; but the New has demonstrated its correctness by actual observation.

As before mentioned, soft gold was entirely used for filling purposes, besides we used also tin, lead, amalgam and gum mastic. The preparations of gold and tin were probably as good as can be produced to day. The amalgam was very much inferior to that now manufactured. The lead fillings were on a par or worse than the copper amalgam used so extensively a few years ago. The gum mastic was for temporary use, now supplanted by wax and gutta-percha.

Separation for filling formally required ten or twelve days, now it is done in as many minutes. Root filling seldom received any attention. The tooth generally receiving the key or forcep after pulp exposure. Porcelain crowns were known as pivot teeth. Of gold crowns we find no mention fifty years ago. Pyorrhea alveolaris was known as scurvy of the gums, and is mentioned by that name at the present time by some dentists.

Operating chairs were large and clumsy, the Wilkinson being the first really improved chair, made about the year 1870, and has no superiors if any equals at the present time.

Now gentlemen, to go on and mention all that might be said about the Old and the New, would take more time than would be just or proper. In closing, let me say, we still live in a progressive age, and to keep abreast with the times, I would advise every practicing dentist to unite with some good dental society; and I know of none better than the Southwestern Michigan—become an active member, give us your papers and your voice in the discussions, and then qualify yourself by remembering the Old and always keeping in touch with the New in your profession.

DISCUSSION.

Dr. Siddell: The question of advertising is of considerable interest in thinking of the old and new. It would be a matter of gratification if we could feel that the new had become more professional and transacted business on more ethical principles. Formerly, at least forty or fifty years ago, we are told it was not customary for one dentist to visit another, and should he do so, everything that was in process of manufacture was studiously concealed from the visitor. Dentists of that time, as a rule, were not anxious to tell of their wonderful successes in the dental societies, or to throw open to every visitor the secrets of their offices and processes.

Dr. Rix: I am an old timer, and I want to say that I always made bold to visit every dentist I could reach, and I tried to find out not what he was doing, but how he did it. I never met a rebuff in any dental office in my life. There was no such flagrant advertising in the old time as we now see all about us. Dental societies came largely into existence when vulcanite was introduced for plates. It brought dentists together to compare notes; it also brought a lot of incapable practitioners into the profession, and it hastened, as the essayist has said, the enactment of State laws regulating practice.

Old men are liable to be egotistical; I hope I am not. I sometimes think the sacrifices made by the pioneers of our profession are not appreciated as they should be. The old does not get the credit it deserves. The new men think they know it all because they get it from the colleges and the books. Ought not the old to receive the credit it deserves for the colleges and the data necessary to make the books?

Dr. Rowley: I think it was Dr. Watling who is responsible for the subject I have presented to-day by a remark he made at our meeting last year. He said, that although he felt as capable of practicing dentistry as he did thirty years ago, yet he felt sure he was losing former patients because of his gray hair. Our young men ought to be more capable, but are they?

Dr. Ellsworth: I began practice in 1861, and have continued steadily at it ever since. It is practically impossible for those who come into the profession to-day to appreciate our disadvantages, and when I compare the then and the now, I feel that much is due to the past, and that we have a right to expect much of the present and still more of the future.

Dr. Hoff: I don't know that the young practitioner, especially the newly graduated, needs any word of commendation; he usually is able to assert himself, and like the newly born bumble bee, "is biggest when hatched." But I think the graduate of to-day is, and he should be, much better prepared to engage in practice than the man who has not had the advantage of a systematic college training, where his course has been laid out for him, and he has had the constant supervision of men who have trained themselves to impart the essential principles of a liberal professional education. The college man is shielded from many faulty doctrines and his mental horizon is broadened, and because of the modern technical methods he should obtain the best manipulative habits.

Then again, the young man, or the practitioner of to-day, ought to do better work from another standpoint. He has the advantage of the older men physically. His youthful vigor and ambition, when properly directed, ought to count for much. His eyesight is keen, his back is suple, his fingers sensitive and agile, and if he knows how, much more ought to be expected from him. And if, in the joy of his youth and vigor, he seem to think that he has all the skill and ability, where is the harm? This should not make us older practitioners envious; and because our eyesight must be helped out by the occulist, and our sense of touch is not as keen as it used to be, or our back is too stiff to allow us to make the large contour gold fillings we formerly did, we should not rob our more fortunate competitors of their opportunity. We can console ourselves with the thought that our judgment is better and more mature than theirs, and that we are still capable of doing the public a good service. And should Dr. Rix's and Dr. Watling's gray hairs drive away some of their younger patients, they should be content, as they have all that they can properly care for left.

Dr. Rix: I would not disparage the efforts of the young man; but I want to say here that I have never given myself to dissipation of any kind, and my eyesight is as good as any man's, and my nerve is as steady, and my years of experience have qualified me as no college can or could have done.

I don't know but that I am the inventor of the rubber dam. I once had, before the rubber dam was suggested, a labial cavity on an incisor tooth that I was unable to keep dry, and finally succeeded in managing it by cutting off a piece of rubber tubing and slipping it over the tooth and forcing it up under the gum, so that I had freedom from moisture and perfect access.

Dr. Watling: I am old enough and unfortunate enough to require the help of the occulist, but I defy any young man to see defects in fillings which I can not find with my glasses. I notice also that students are not entirely free from backache. Physical well being is no doubt of the greatest value to a practioner, but experience is of infinitely more value. The experienced practitioner will make, to begin with, a more accurate diagnosis and he will not have to do so much experimenting in treatment. There is no doubt that many of our younger men can make difficult operations, and with much skill and credit to themselves and the profession; but too many of our young men do not, or will not, learn to persistently and continually do the best that they are capable of. It is the older practitioner only, who from his long experience, knows that every operation must be done as well as it is possible to make it.

There are many aids and appliances to be had to-day that were not avilable in my early experience. If I were asked to name the one that should be given the honor of first place, I think I should have to give it to the rubber dam; it is absolutely essential for the practice of to-day.

Dr. Runyan: I do not think the cause for the loss of patients by the older practitioners is due to the fact that the public loses confidence in either the skill or ability of the old men. But it is too often due to social relations. It is the young people, between the ages of twelve and thirty, who have the most operating to be done, and these are the associates, socially, of the young dentist, and they naturally apply to them for professional services, in spite of the fact that their fathers and mothers continue with the older men.

